# Epidemiology of glucose-6-phosphate dehydrogenase deficiency in 10 malaria-endemic countries in sub-Saharan Africa: a systematic review

**DOI:** 10.11604/pamj.2025.50.7.45066

**Published:** 2025-01-06

**Authors:** Mariem Taleb, Aminetou Taleb Brahim, Cheikh Yebouk, T'feila Khairy, Beybeti Ishagh, Badiaa Lyoussi, Sidi Mohamed Ghaber

**Affiliations:** 1 Laboratory of Natural Substances, Pharmacology, Environment, Modeling, Health, and Quality of Life (SNAMOPEQ), Department of Biology, Faculty of Sciences Dhar El Mahraz, University Sidi Mohamed Ben Abdallah, 30000 Fez, Morocco,; 2 Department of Molecular Research, Maurilab Medical Analysis and Research Institute, 2434 Nouakchott, Mauritania,; 3 Unité de Recherche Génomes et Milieux, Faculté des Sciences et Techniques, Université de Nouakchott, Nouveau Campus Universitaire, BP 5026, Nouakchott, Mauritanie,; 4 Department of Biology, Laboratory of Plant Biodiversity and Natural Resource Development, University of Nouakchott, Nouakchott, Mauritania,; 5 Unité d´Epidémiologie Moléculaire et Diversité des Microorganismes, Faculté des Sciences et Techniques, Université de Nouakchott, Nouakchott 2373, Mauritanie,; 6 Faculté de Médecine, de Pharmacie et d´Odonto-stomatologie, Université de Nouakchott, Nouveau Campus Universitaire, BP 880 Nouakchott, Mauritanie

**Keywords:** Wasting, growth monitoring, mid-upper arm circumference, infant and young child feeding, Africa

## Abstract

**Introduction:**

it is now clear that several antimalarials, such as primaquine, can cause severe hemolytic anemia in people with G6PD deficiency. Prescriptions for antimalarials should consider G6PD status, especially in malaria-endemic areas. The frequency of G6PD is quite high in some countries in sub-Saharan Africa.

**Methods:**

a systematic review was conducted to summarize the findings on the G6PD deficiency epidemiology in the ten African nations most impacted by malaria. We conducted a systematic electronic literature search based on eligibility requirements using databases, PubMed, Google Scholar, and ScienceDirect. The chosen studies are original or primary studies published in English or French and evaluate the prevalence of G6PD deficiency or its incidence, in a peer-reviewed scientific journal during the interval of time 2000-2023 were included in this review. The systematic review was carried out by using PRISMA (Preferred Reporting Items for Systematic Reviews and Meta-Analyses).

**Results:**

after being screened, 19 complete texts were deemed appropriate for extracting data. The prevalence of G6PD deficiency varied among these ten African countries, ranging from <1% to 23.9%, with a considerable dominance when it comes to men in comparison with women.

**Conclusion:**

to avoid morbidity and mortality among the population in the affected areas, several regions in African countries must review their diagnostic and screening protocols to detect deficiency in G6PD to make proper interventions in time.

## Introduction

Ten percent (10%) of the energy required by red blood cells is produced by anaerobic glycolysis, which is facilitated by enzyme key the glucose-6-phosphate dehydrogenase for the regeneration of reduced glutathione. Reduced glutathione is necessary for red blood cell protection against oxidation and plays a critical role in the regulation of oxidative stress [1].

The deficit of G6PD is a hereditary disease, the gene responsible for this condition is carried by the X chromosome, more precisely on the long arm of the Xq28 [2]. Its transmission is therefore linked to sex, and most subjects are asymptomatic [3]. Men are mostly affected because they have only one X. Homozygous women and hemizygous men are always symptomatic. Females that transmit the anomaly are usually unharmed [4]. It can cause serious problems, especially if G6PD-deficient erythrocytes are exposed to different exogenous triggers, particularly in the treatment of malaria [2]. In the presence of reduced enzyme activity, oxidizing agents can denature hemoglobin and membrane lipids, promoting red blood cell lysis [5].

Acute hemolytic crises in patients with G6PD deficiency can be triggered by the use of some antimalarial medications (primaquine, tafenoquine), ingestion of a specific type of bean (fava beans), and a variety of infections (typhoid fever, hepatitis, malaria). These crises can range in severity, and in certain situations, an emergency blood transfusion may be necessary [6]. In newborns, G6PD deficiency is one of the main causes of hyperbilirubinemia, which can potentially cause nuclear jaundice [7]. Based on epidemiological data, G6PD deficiency is the most prevalent enzymopathy globally, impacting around 500 million people [5]. This deficiency is widespread in Africa, the Mediterranean, the Middle East, and Southeast Asia [8], and this enzymopathy could reach 32.5% in sub-Saharan Africa [9]. It is very common in areas where *Plasmodium falciparum* infection is endemic [9]. Several studies have established a connection between G6PD deficiency and malaria, suggesting that the deficit offers some defense against the disease's most severe forms [10,11]. On the other hand, the use of antimalarials can cause iatrogenic hemolytic crises in patients with G6PD deficiency [12].

According to the WHO, 11 countries worldwide are the most affected by malaria, of which 10 are in Africa (Democratic Republic of the Congo (DRC), Burkina Faso, Ghana, Cameroon, Mali, Mozambique, Niger, Nigeria, Uganda and United Republic of Tanzania) [13]. These ten African countries must therefore be informed of the true prevalence of enzyme deficiency, which will allow appropriate decisions to be made concerning the potentially hazardous usage of antimalarial medications for patients suffering from G6PD deficiency. However, the objective of this systematic review is to assess the prevalence of G6PD deficiency in the 10 malaria-endemic countries in sub-Saharan Africa.

## Methods

**Study design:** we conducted a systematic review following the Preferred Reporting Items for Systematic Reviews and Meta-Analyses (PRISMA) guidelines [14].

**Eligibility criteria:** research studies that evaluated the incidence or prevalence of glucose-6-phosphate dehydrogenase deficiency that were published in peer-reviewed scientific publications between 2000 and 2023 in either English or French were included in this review. We excluded case reports, editorials, reviews, and conference abstracts without primary data.

**Data synthesis:** two authors (A.T.B. and B.I.) independently reviewed and screened titles and abstracts. Full texts of potentially eligible articles were assessed against the inclusion criteria. A third reviewer (M.T.) resolved disagreements. Extracted data included author(s), publication year, study location, sample size, participant demographics (age, sex, ethnicity if available), diagnostic methods, and prevalence estimates.

**Source of information and search strategy:** electronic databases were used in this systematic electronic literature such as Google Scholar, PubMed, and ScienceDirect after using the eligibility criteria mentioned in the “Eligibility criteria” section. The search of these databases was carried out using a pre-established search strategy and was conducted using MeSH terms, keywords, and combinations of these words. A series of search phrases were used to find research publications, and the combinations of search terms were chosen according to the research topic posed. Three primary concepts, deficiency of Glucose-6-Phosphate Dehydrogenase, epidemiology, and country names have been established. Boolean operators (AND, OR) were used to combine some of the very important key concepts. Such as (“Epidemiology)”, AND “Glucose-6-Phosphate Dehydrogenase deficiency”, OR “G6PD deficiency” AND “Burkina Faso”, “Cameroon”, “the Democratic Republic of the Congo (DRC)”, “Ghana”, “Mali”, “Mozambique”, “Niger”, “Nigeria”, “Uganda and “the United Republic of Tanzania”). Each database's search technique was customized, and the same keywords and Boolean descriptors were employed across all of them.

**Figure 1:**
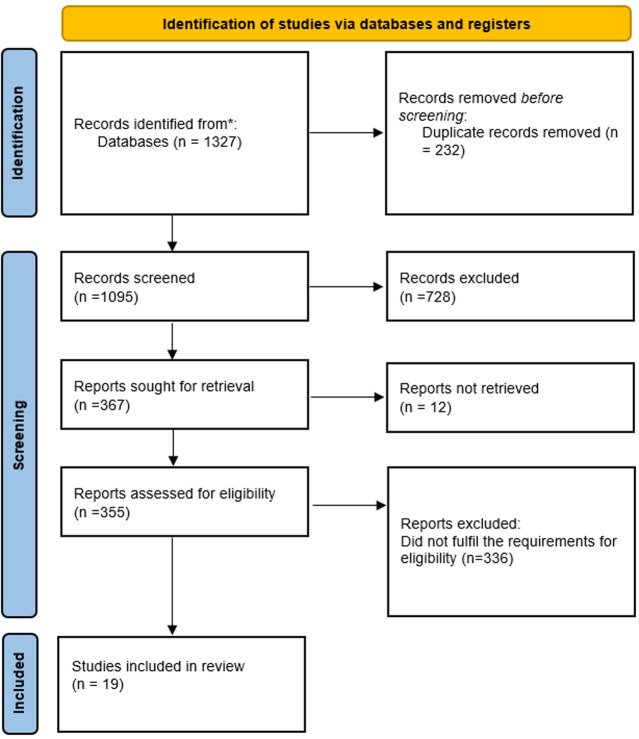
flowchart summarizing the process of identifying and selecting papers for a systematic review

**Selection of studies:** the bibliographic management program Zotero version 6.0.36 was utilized to arrange every research article that was exported from the databases. Duplicate items were eliminated and folders were made for PubMed, Google Scholar, and ScienceDirect databases. In Zotero, unique research from the databases was first arranged by title. Then, eligibility was ascertained by looking at the abstracts of the papers that made the shortlist using the predetermined standards. Ultimately, the whole manuscripts of the accepted articles underwent examination. The whole procedure of selecting the final eligible research papers is depicted in the PRISMA diagram (Figure 1).

**Quality appraisal:** we critically appraised the included studies by assessing sample representativeness, diagnostic methodology, clarity of reporting, and risk of bias (e.g., non-random sampling). Differences in testing methods and study populations were noted to contextualize variability in reported prevalence.

## Results

From the databases, 1,327 articles in total were gathered. 1,095 articles of them were found to be pertinent to the subject and were looked at after 232 duplicates were removed. After 367 full-text publications were evaluated for eligibility, 348 of them were found to be ineligible. As a result, only 19 articles were found to be qualified and were added to the document.

**Characteristics of the included studies:** as indicated in Table 1, research was carried out in many nations, including one study from each Mali, Mozambique, and the Democratic Republic of the Congo (DRC); two studies from Nigeria and Uganda; and three studies from Burkina Faso, Cameroon, Ghana, and the United Republic of Tanzania. With respect to the dates of publication, there were five studies published from 2000 to 2010, six studies from 2011 to 2015, five studies from 2016 to 2020, and three studies from 2020 to 2023. The total sample size was 28,549 individuals, with varying gender distributions and G6PD deficiency prevalence rates. All included studies employed valid and reliable screening and diagnostic methods for detecting G6PD deficiency. As detailed in Table 2, four studies utilized quantitative enzymatic assays, the reference standard for diagnosing G6PD deficiency. Six studies applied the fluorescent spot assay, a widely used semi-quantitative method that is rapid, inexpensive, reliable, and easy to perform. Additionally, nine studies employed molecular approaches to detect specific mutations, commonly used for population screening, family studies, or prenatal diagnosis.

**Table 1:** epidemiology of G6PD deficiency based on the studies included in the review (n=19)

Authors	Year	Country	Sample size	Target population	Age	Prevalence (%)
Simpore *et al*.	2007	Burkina Faso	342	7 villages and 10 elementary schools	7-32 years	16.3%
Ouattara *et al*.	2014	Burkina Faso	200	Individuals from a rural community	1-79 years	9.5%
Ouattara *et al*.	2016	Burkina Faso	182	Patients tested positive for malaria	1-72 years	9.9%
Uzoegwu and Awah	2006	Cameroon	12470	Individuals from Northwest and Southwest	1-70 years	11.61%
Awah and Uzoegwu	2008	Cameroon	606	Volunteers	3-70 years	10.4%
Lauden *et al*.	2019	Cameroon	1512	Blood donors	≥18 years	7.9%
Amoah *et al*.	2016	Ghana	170	Students in Ghana	5-12 years	10.6%
Adu *et al*.	2016	Ghana	200	Blood donors	≥18 years	19.5%
Amoah *et al*.	2021	Ghana	6108	Study in 10 health facilities	Not specified	20.06
Maiga	2014	Mali	712	Two ethnic groups Dogon and Fulani in rural village Manteourou	Not specified	Fulani= <1%; Dogon=7.7%
Galatas *et al*.	2017	Mozambique	2070	School children from 12 schools in 3 different regions of Mozambique	Average age 17.9	Pemba=8.3%; Manhiça=14.6%; Mocuba=16.8%
Ademowo and Falusi	2002	Nigeria	721	Homogeneous population of Yoruba tribe in southwest Nigeria	Children 0.6-12 years 15-56 years	23.9% among man; 4.6% among women
Williams *et al*.	2013	Nigeria	1122	Children from different ethnic groups in Nigeria	1 month -15 years	15.3%
Fanello *et al*.	2023	RCD	365 NN et 362 mothers	Newborns	NN	8.3% among NN; 2.2% among mothers
Segeja *et al*.	2008	Tanzania	415	Random samples from Muheza, a malaria endemic area in Tanzania	6 months -45 years	Lowlands 11.32%; Highlands 4.43%
Mwakasungula *et al*.	2014	Tanzania	384	Unrelated volunteers living in Dar es Salaam	Not specified	G6PD (A) 16.4 %; G6PD (A-) 13.5 %
Manjurano *et al*.	2023	Tanzania	263	Individuals northwest in Tanzania	0-87 years	16.4%
Bwayo *et al*.	2014	Uganda	245	Children living in Iganga, eastern Uganda	6 months-9 years	20.41%
Hsu *et al*.	2014	Uganda	100	Newborns	Newborns	6%

**Table 2:** diagnostic techniques for G6PD deficiency reported in the included studies (n=19)

Authors	Year	Country	Diagnostic technique	Malaria
Simpore *et al*.	2007	Burkina Faso	Enzymatic test	Asymptomatic
Ouattara *et al*.	2014	Burkina Faso	Real-time PCR	Asymptomatic
Ouattara *et al*.	2016	Burkina Faso	PCR Classique	Symptomatic
Uzoegwu and Awah	2006	Cameroon	Fluorescent spot test	Asymptomatic
Awah and Uzoegwu	2008	Cameroon	Fluorescent spot test	Symptomatic
Lauden *et al*.	2019	Cameroon	Fluorescent spot test	Asymptomatic
Amoah *et al*.	2016	Ghana	PCR/RFLP	Asymptomatic
Adu *et al*.	2016	Ghana	Enzymatic test	Asymptomatic
Amoah *et al*.	2021	Ghana	PCR/RFLP	Symptomatic
Maiga	2014	Mali	Genotyping by Sequenom; MassARRAY IPLEX	Asymptomatic
Galatas *et al*.	2017	Mozambique	Enzymatic test	Asymptomatic
Ademowo et Falusi	2002	Nigeria	Enzymatic test	Asymptomatic
Williams *et al*.	2013	Nigeria	Fluorescent spot test	Asymptomatic
Fanello *et al*.	2023	RCD	Fluorescent spot test and CareStart; G6PD	Asymptomatic
Segeja *et al*.	2008	Tanzania	Fluorescent spot test	Asymptomatic
Mwakasungula *et al*.	2014	Tanzania	PCR/Sequencing	Asymptomatic
Manjurano *et al*.	2023	Tanzania	PCR/RFLP	Asymptomatic
Bwayo *et al*.	2014	Uganda	PCR/RFLP	Asymptomatic
Hsu *et al*.	2014	Uganda	Real-time PCR	Asymptomatic

**Epidemiology of G6PD deficiency in malaria-endemic countries: main findings:** prevalence estimates ranged from <1% in parts of Mali to as high as 23.9% in Nigeria. Simpore *et al*. studied 342 participants, ages 7 to 32, in Kadiogo, Burkina Faso. The overall prevalence of G6PD deficiency was 16.3% (56/342). The authors noted that the prevalence increases with age 6-9 years (8.4%); 10-19 years (20.9%) and 20-29 years (25%) and also that men are more affected than women with a prevalence of 20.5% and 12.3% respectively with P=0.041 [15]. Ouattara *et al*. conducted a study on 200 subjects living in a rural area in Burkina Faso, aged 1 to 79, this population is composed of 58% women and 42% men. The prevalence of G6PD deficiency is 9.5% in this population. The authors found that the prevalence of G6PD deficiency was significantly higher in males than females (14.3% vs. 6.0%, P = 0.049) [16]. The G6PD A- variant (*A376G /202A*) is the only variant detected with a prevalence of 9.5% (19/200) and none of the following variants *A376G/542T, A376G/680T*, and *A376G/968C* have been detected in this population [16]. Ouattara *et al*. conducted a prospective study in 2016 in three health centers in Ouagadougou, Burkina Faso. The study involved 182 patients aged 1 to 72 years with a mean age of 17.1 ± 13.9 years. The study population consisted of 50.5% (92/182) males and 49.5% (90/182) females. The prevalence of G6PD deficiency is 9.9% (18/182). The authors found that the prevalence of hemizygous men was significantly higher than that of homozygous women (15.5% vs. 4.4%, p=0.015) and that the frequency of the HbS allele was 0.014 vs. 0.126 for the HbC allele [17]. Factors influencing this heterogeneity likely include differing genetic backgrounds, local selective pressures from malaria, and historical migration. For instance, in Ghana and Nigeria.

Uzoegwu and Awah conducted a study in 12470 samples from the North-West and South-West of Cameroon aged between 1-70 years. The prevalence of G6PD deficiency was 11.61% in this population. The authors found that men are more affected than women with prevalences of 9.21% and 1.20% respectively and that the prevalence of the deficit in the population of the North-West is 9.21% and 10.85% in the South-West [18]. Awah and Uzoegwu conducted a cohort of 606 volunteers from Cameroon, aged between 3 and 70 years. The prevalence of G6PD deficiency was 10.4%. The authors found that men are more affected than women with a respective prevalence of 9.2% and 1.2 with a p-value of <0.05 [19]. Lauden *et al*. conducted a cross-sectional study of 1512 blood donors in Cameroon, the majority of donors were male 74% (n=1001). The authors found that the prevalence of G6PD deficiency in blood donors is 7.9%. The authors did not find a significant difference (p = 0.27) between the mean age of G6PD-deficient blood donors and G6PD-deficient donors who are 32.8 years and 31.6 years of age, respectively. Same thing, no significant difference (p=0.52) in the assessment of hemoglobin concentration (Hgb) between normal donors in G6PD (14.8 g/dL) and donors deficient in G6PD (14.9 g/dL) [20].

Amoah *et al*. conducted a longitudinal study in 170 students in southern Ghana aged between 5-12 years, of whom 10.6% are phenotypically deficient in G6PD. The authors performed G6PD genotyping, they found that only one sub-Saharan allele was detected in this population with a prevalence of 12.4% (21/170) [21]. Adu *et al*. conducted a cross-sectional study in Berekum, Ghana in 200 blood donors. Glucose-6-phosphate dehydrogenase deficiency is 19.5% prevalent, the authors showed that 7% of donors had both a complete qualitative defect of the G6PD enzyme and a hemoglobin AS phenotype, 12.5% of donors had only the hemoglobin AS phenotype [22]. Amoah *et al*. in 2021 conducted a cross-sectional study on 6108 subjects collected from 10 health facilities in 10 regions of Ghana. The results of this study showed that G6PD genotypic variants were homogeneous across Ghana with a prevalence of 20.06% (1225/6108) of G6PDd variant A (including homozygous and heterozygous females, hemizygous men). The authors showed that their results show that homozygous deficient female AA- , hemizygous male A-, heterozygous female BA- , and AA- G6PD variants protect against malaria infections in all study regions [23]. Maiga *et al*. conducted a cross-sectional study of 712 subjects from two different ethnic groups, Dogon and Fulani, living in the rural village of Manteourou in Mali. The Dogon ethnic group accounts for 52.7% (375/712) of the population studied and Fulani 47.3% (337/712). The allelic frequency of G202A is 7.7% in the Dogon while the Fulani is <1%, for the Betica-Selma variant *A376G/968C* is 6.1% in the Fulani ethnic group while it is absent in the Dogon. The authors demonstrated that, compared to Dogons, Fulani are less susceptible to malaria [24].

Galatas *et al*. conducted a cross-sectional study of school-based students from twelve schools in three geographically different areas in Mozambique which are Manhiça, located in the south; Mocuba in the center and Pemba at the northern tip of the country. The average age of the participants is 17.9 years. The authors found that the prevalence of G6PD varies within the country, the highest prevalence was observed in the Mocuba center (16.8%) followed by Manhiça (14.6%) in the south and the lowest prevalence is in Pemba (8.3%) located in the north of the country. Glucose-6-phosphate dehydrogenase deficiency is more common in boys (10.6%) than in girls (6.5%) with (p = 0.03). Mocuba and Manhiça girls showed lower prevalence (14.7% and 14.1%) respectively, in contrast to boys (18.9% and 15.4%) respectively [25]. Ademowo and Falusi conducted a study in a homogeneous population in Nigeria, this study was carried out on 721 members of the Yoruba tribe from southwestern Nigeria, these subjects were divided into two groups of children aged between (0.6-12 years and adults 15-56 years), with 373 men and 348 women. The samples underwent enzymatic assay, the authors found that the prevalence of G6PD deficiency was 23.9% and 4.6% in males and females, respectively. And that only the G6PDA- genotype was found in this homogeneous population [26]. Williams *et al*. conducted a study on 1122 children in Nigeria (561 boys and 561 girls) the children participating in the study were between 1 month and 15 years old. Fifteen-point-three percent (15.3%) of study participants were deficient in G6PD, males had a higher prevalence (24.1%) than females (6.6%), the authors found that Yoruba children are the most affected with a prevalence of (16.9%) than children from Igede (10.5%), Igbo (10.1%) and Tiv (5.0%). And that only the G6PDA- genotype was found in this homogeneous population [27].

Fanello *et al*. conducted a hospital-based cohort in a semi-rural area of Kinshasa (Democratic Republic of Congo) in 362 mothers with 365 newborns. The prevalence of G6PD in newborns is 8.3% (29/349) the allele frequency of G6PD A- was 14.6% in males and 15.5% in females. The prevalence of G6PD deficiency among mothers was 1.9% according to the FST and 2.2% according to the RDT. The authors found hyperbilirubinemia which occurred in 5.7% of cases during the first 3 days of life [28]. Segeja *et al*. conducted a cross-sectional study in lowland and mountain areas in Muheza, Tanzania, in 1959 subjects aged between 6 months and 45 years, of whom 415 were screened for G6PD deficiency. Lowlands have a higher frequency of Hbs and G6PD deficiency than highlands. (G6PD deficiency = 11.32% (24/212) vs. 4.43% (9/203), P = 0.01 and HbS = 16.04% (98/611) vs. 6.32% (36/570), P = 0.0001) [29].

Mwakasungula *et al*. performed research on 384 healthy, randomly chosen, unrelated male volunteers from Tanzania who lived in Dar es Salaam. In this population, the deficiency of G6PD prevalence was 16.4% (63/384) and 13.5% (52/384) of hemizygous volunteers for the enzymopathies G6PD A and G6PD A- respectively. The authors showed that the most important red blood cell polymorphism was heterozygous α^+^-thalassemia (37.8%), followed by G6PD A deficiency (16.4%), heterozygous sickle cell trait (15.9%), and G6PD A- deficiency (13.5%), and homozygous α^+^-thalassemia (5.2%) [30]. Manjurano *et al*. conducted a retrospective cross-sectional study of 263 subjects in Misungwi located in the Mwanza region of northwestern Tanzania, of whom 145 (55.1%) were women. The subjects are aged between 0-87 years, the median age is 14 years. The prevalence of G6PD deficiency in this population is 16.4%. The authors found that the prevalence of the A- mutation is 8% (21/263) in heterozygous women and 8.4% (22/263) in hemizygous or homozygous. The authors found that 58.1% (144/248) showed positive PCR results for *P. falciparum* [31].

Bwayo *et al*. conducted a longitudinal study of 245 children in Iganga, Uganda. These children are between 6 months and 9 years old, the average age is 4.4 (±2.3). The prevalence of G6PD A- deficiency is 20.4% (50/245). The authors showed that the prevalence of asymptomatic *Plasmodium falciparum* infection is 40.8% (100/245) in this study population [32]. Hsu *et al*. conducted a study on 100 newborns at Mulago Hospital in Kampala, Uganda. Umbilical cord samples were analyzed, including 50 males and 50 females. The overall allele frequency of *G202A* was 0.13 and that of *A376G* was 0.32. The authors found that the overall prevalence of the A- variant (*A376G /202A*) in this study population is 6% [33].

## Discussion

This systematic review was conducted to determine the prevalence of G6PD deficiency in ten malaria-endemic countries located in sub-Saharan Africa. The result obtained in this study suggests that the prevalence of G6PD deficiency is very heterogeneous and extremely variable, from one country to another, but also within the same country. The prevalence found in this study ranges from <1% in parts of Mali to as high as 23.9% in Nigeria. First, we can explain this heterogeneity in prevalence by the fact that the different surveys were conducted in each country separately and genetic heterogeneity of the population, sample size and composition, environment, and migration history; these factors can introduce variations between different regions, modifying the distribution of G6PD deficiency. The second difference may be due to screening techniques that differ from one study to another.

Glucose-6-phosphate dehydrogenase is the central enzyme in the red blood cell antioxidant cascade [34]. It allows the synthesis of NADPH necessary for the production of reduced glutathione within the pentose pathway. Glutathione detoxifies the cell from hydrogen peroxide molecules through glutathione reductase [10], which is a mechanism for protecting red blood cells from oxidative damage. Red blood cells that are deficient in G6PD are very fragile, which facilitates their hemolysis. Globally, the prevalence of G6PD deficiency varies from region to region [35,36]. In general, G6PD deficiency is the most common enzymopathy in the world, according to the literature, sub-Saharan Africa contains areas that are the most affected with the highest prevalence [36].

In this review, men are more affected by G6PD deficiency than women, and this is consistent with all studies conducted around the world [37]. This pathology generally affects (90%) males [38]. Glucose-6-phosphate dehydrogenase deficiency is an inherited sex-related condition, as it originates from an abnormal gene on the X chromosome. Since the majority of genes associated with the X chromosome undergo the phenomenon of X chromosome inactivation, heterozygous women are epigenetic mosaics in their red blood cells which causes a mixture between normal red blood cells and G6PD-deficient red blood cells [5], heterozygous women transmit normally, but without risk of pathology, due to the phenomenon of lionization [39]. Homozygous women and hemizygous men fully express this deficiency [4].

Despite the difference in screening and diagnosis strategies, which vary from one geographical area to another, according to the recommendations of the World Health Organization, each country where the prevalence of deficiency is between 3 and 5% in men must carry out systematic screening of newborns [40]. As a result, malaria-endemic African countries where men are the most affected by G6PD deficiency will need to demonstrate a national newborn screening program, according to the WHO, the estimated number of deaths attributable to malaria rise to 608,000 in 2022 compared to 610,000 in 2021. About 95% of deaths associated with this disease were recorded in Africa during 2022. In this region, over 78% of malaria-related fatalities occurred in children under the age of five [41]. In malaria-endemic countries, estimates of the prevalence of G6PD deficiency resemble those found in malaria-endemic countries [42,43]. It is then necessary to test for G6PD before prescribing antimalarials to avoid hemolysis in G6PD-deficient patients.

Vulnerable groups are affected by G6PD deficiency, including children under five years old, who are more susceptible to infections that can cause severe hemolytic crises, necessitating early screening measures and parent education programs. Newborns and mothers who are expecting, because a pregnant woman with the G6PD gene can pass the defect to her offspring and the afflicted infant is more likely to have severe jaundice, prenatal and neonatal screening is essential in avoiding difficulties in both pregnant women and newborns [44]. Untreated crises are more likely to occur in rural and low-income areas because these groups frequently have less access to medical treatment and diagnostic testing. To increase these people's access to knowledge and treatment, particular awareness-raising is needed.

These countries can manage hemolytic anemia effectively if it is diagnosed early, with trustworthy diagnostic techniques being available, and in situations where antimalarial medications are required to eradicate malaria, point-of-care testing becoming increasingly crucial. Therefore, early detection of this pathology and care of newborns can help these countries reduce the mortality rate and the development of nuclear jaundice in children. In addition, after detecting the deficiency of G6PD in time, the health issues associated with antimalarial medication usage and transfusion risk can be resolved.

This review provided information on the epidemiology of G6PD deficiency in the 10 malaria-endemic countries in sub-Saharan Africa. PRISMA criteria were followed in conducting this evaluation, which examined data from 19 research studies. The absence of research in some of these countries has meant the 10 countries were not equally represented. The findings can help doctors, pediatricians, and health authorities understand the importance of regular newborn screening in identifying G6PD deficiency early on and enhancing health outcomes.

The necessity of specific public health policy is highlighted by the results presented above. G6PD testing should be integrated into standard malaria diagnostic services or newborn screening should be implemented in high-prevalence areas. In order to provide antimalarial medications like primaquine in a safer manner, healthcare practitioners need to be trained on when to do tests and how to interpret G6PD findings. More selective tactics, including community awareness campaigns or subgroup screening, could be suitable in low-prevalence environments. Point-of-care G6PD testing equipment can help avoid hemolytic consequences and speed up diagnosis, especially in places with limited resources or distant locations. To fully understand the genetic variants that are common in various places and how variables like ethnic makeup, climatic variations, and the capacity of the health system affect prevalence, more studies are required. Longitudinal research examining the effects of interventions (such as screening initiatives, provider education, and policy modifications).

## Conclusion

In this systematic review, these ten countries have high and variable rates of G6PD deficiency in sub-Saharan Africa, a region heavily burdened by malaria. Men are more likely than women to have it, according to one study. To reduce iatrogenic hemolytic episodes and morbidity and mortality, it will be essential that different regions within these countries update their screening and diagnostic procedures for G6PD deficiency before administering antimalarials, particularly for children under five. while increasing health practitioners' awareness. It would be beneficial to carry out nationwide standardized studies that take into consideration ethnic variation in each nation and employ dependable, consistent screening techniques. Adopting specialized public health strategies that take into account regional prevalence, genetic origins, and healthcare infrastructure is essential to reduce treatment-related risks and enhancing health outcomes. It will be crucial to integrate G6PD testing into malaria prevention efforts, train healthcare workers, and establish screening procedures. Going ahead, iatrogenic hemolysis may be decreased and the safety and efficacy of malaria eradication efforts can be increased by comprehending the genetic landscape and putting context-specific therapies into practice.

### What is known about this topic

- G6PD deficiency is a common enzymopathy worldwide, with significant regional variability; it is particularly prevalent in sub-Saharan Africa, the Middle East, and Southeast Asia;
- Impact on red blood cells: G6PD is crucial for red blood cell protection against oxidative damage; its deficiency leads to fragile red blood cells prone to hemolysis, especially under oxidative stress;
- G6PD deficiency mostly affects men; hemizygous men and homozygous women completely display the deficit, but heterozygous women are usually asymptomatic carriers.

### What this study adds

- This study offers current information on the diverse prevalence of G6PD deficiency in ten sub-Saharan African nations where malaria is widespread;
- The study supports the need for gender-specific screening methods by confirming that males are more likely than women to be afflicted by G6PD deficiency; this finding is consistent with worldwide trends;
- According to the study's molecular results, the A- variant (A376G/202A) is the most prevalent in these ten sub-Saharan African countries where malaria is endemic; prenatal and neonatal screening in malaria-endemic countries is essential to prevent the development of hemolytic anemia in both pregnant women and newborns, thereby addressing health issues related to the use of antimalarial drugs and the risks related to transfusions.
